# Why Do Mothers of Young Infants Choose to Formula Feed in China? Perceptions of Mothers and Hospital Staff

**DOI:** 10.3390/ijerph120504520

**Published:** 2015-04-24

**Authors:** Ke Zhang, Li Tang, Hong Wang, Li-Qian Qiu, Colin W. Binns, Andy H. Lee

**Affiliations:** 1 Women’s Hospital, School of Medicine, Zhejiang University, Hangzhou, Zhejiang 310006, China; E-Mail: zhangke@zju.edu.cn; 2 Department of Management Sciences, City University of Hong Kong, Hong Kong 999077, China; E-Mail: li.tang@curtin.edu.au; 3 School of Public Health, Curtin University, Perth, WA 6845, Australia; E-Mails: c.binns@curtin.edu.au (C.W.B.); andy.lee@curtin.edu.au (A.H.L.); 4 Shenzhen Maternity and Child Healthcare Hospital, Shenzhen, Guangdong 518038, China; E-Mail: xwanghong@163.com

**Keywords:** breastfeeding, infant formula, qualitative research, China

## Abstract

In China the exclusive breastfeeding rate remains low and infant formula is widely used. This study aimed to elicit and compare mothers’ and hospital staff perceptions of the reasons that shaped mothers’ decision to formula feed. In-depth interviews with 50 mothers, and four focus group discussions with 33 hospital staff, were conducted in Hangzhou and Shenzhen in November 2014. Responses given by the mothers and hospital staff showed a number of commonalities. The perception of “insufficient breast milk” was cited by the majority of women (n = 37, 74%) as the reason for formula feeding. Mothers’ confidence in breastfeeding appears to be further reduced by maternal mothers or mothers-in-law’s and “confinement ladies” misconceptions about infant feeding. Inadequate breastfeeding facilities and limited flexibility at their workplace was another common reason given for switching to formula feeding. A substantial proportion of mothers (n = 27, 54%) lacked an understanding of the health benefits of breastfeeding. Antenatal education on breastfeeding benefits for expectant mothers and their families is recommended. Moreover, mothers should be provided with breastfeeding support while in hospital and be encouraged to seek professional assistance to deal with breastfeeding problems after discharge. Employers should also make work environments more breastfeeding-friendly.

## 1. Introduction

Breastfeeding is widely recognized as the optimal feeding method for infants and young children. Breastfed infants are at reduced risk of many health conditions, including gastrointestinal and respiratory tract infections, obits media, allergies, and sudden infant death syndrome [1,2]. Lactation is also associated with many positive long-term health outcomes, such as decreased risks of obesity and diabetes, and increased performance in intelligence tests during childhood and adolescence [1,3]. For mothers, the advantages of breastfeeding include reduced postpartum bleeding, faster return to pre-pregnancy weight, a lower risk of breast and ovarian cancer, as well as decreased risks of hip fractures and osteoporosis in later life [1,4,5].

The many health benefits of breastfeeding have led the World Health Organization to recommend mothers to exclusively breastfeed for the first six months of life and continue while complementary feeds are introduced. However, the exclusive breastfeeding rate in China remains low and infant formula is widely consumed across the country, either as a first feed or as complementary feed [6,7]. A cohort study conducted in Shihezi, Xinjiang Uygur Autonomous Region, found that less than 3% of infants were exclusively breastfed for the first six months when measured prospectively [8]. In Zhejiang Province, over half the infants had already consumed, or were still consuming, some infant formula by one month postpartum; and the proportion reached 98% by six months [9]. Results of our recent survey conducted in Hangzhou and Shenzhen showed that approximately 65% of infants aged within six months had consumed some infant formula by the time of interview [10]. Indeed, China is now the largest infant formula market in the world, with a projected annual growth rate of 20% [11].

Based on previous studies on breastfeeding in China, younger age, maternal return to work, shorter intended duration of breastfeeding and premature introduction of complementary feeds, are common factors associated with early cessation of breastfeeding [9,12]. However, only limited studies have investigated the factors associated with Chinese mothers’ decision to formula feed. A cohort study conducted in Zhejiang Province reported that for births in the city of Hangzhou, late decision to breastfeed and infants being given a pre-lacteal feed were risk factors of formula introduction before three months of age [9]. To the best of our knowledge, no research has examined the reasons of formula usage by mothers in China. Moreover, despite the effort of hospital staff in promoting and supporting breast feeding; there have been no studies that explore the understanding by staff why mothers choose to formula feed.

In view of the disadvantages of formula feeding, the wide spread use of infant formula in China, and the lack of published research, the present study aimed to elicit and compare mothers’ and hospital staff perceptions of the reasons underlying the decision to formula feed over breastfeeding.

## 2. Materials and Methods

### 2.1. Design and Setting

A qualitative study using in-depth interviews of mothers and focus group discussions with hospital staff was conducted in Hangzhou and Shenzhen in November 2014. Hangzhou, with a population of seven million, is the capital city of Zhejiang Province located in Eastern China. Shenzhen is a special economic zone adjacent to Hong Kong and a major financial centre for Guangdong province in southern China. These two relatively affluent cities were chosen because of the widespread use of infant formula in urban areas where women can afford to purchase and the products are readily available. One maternal and child health hospital and two community health centers were chosen in each city, with a total target sample size of 50 mothers and 33 hospital staff.

### 2.2. Participants and Data Collection

The research consisted of two components. In the first part, Mandarin speaking mothers within one to six months postpartum were approached when they brought their infant to the chosen sites for immunization and regular health examinations. Small incentives were provided to encourage participation. Inclusion criteria were mothers aged 18 years or older with a single delivery and had completed junior high school education or higher. Mothers were excluded if the infant was born prematurely (<37 weeks gestation), had a low birth weight (<2500 g), was admitted to the neonatal intensive care unit, or had been exclusively or fully breastfed at the time of interview. 

Fifty eligible mothers (25 in each city) who gave consent were interviewed in private by the first or second author using a semi-structured questionnaire. Demographic information including age at childbirth, educational level, gender of infant, type of delivery and gestational age at birth, was sought before interview. Mothers were then asked a series of questions about their perceptions of the reasons that shape their decision to formula feed, their knowledge on infant feeding, and their sources of information on formula feeding. Core questions remained the same for each mother in order to pursue research themes; however, further ideas and concepts were generated using mothers’ own words and phrases. The length of interviews ranged from 30 to 45 min.

In the second part, two focus group discussions with eight to nine hospital staff per group were conducted at each city. A total of 33 hospital staff (17 from Hangzhou and 16 from Shenzhen) who worked closely with mothers of young infants participated in the discussions. Basic information on age, department, position and years in practice was collected using a brief self-administered questionnaire. An interview schedule composed of eight open-ended questions was used to explore the perceptions and beliefs of hospital staff regarding formula feed by mothers. Each focus group discussion lasted approximated 50 min. 

The guides for these in-depth interviews and focus group discussions were developed after an extensive review of the literature on infant feeding and recommendations from experts. All interviews and discussions were audio tape-recorded upon permission of the participants. Copies of the questionnaire and group discussion guide are available upon request from the authors.

### 2.3. Data Analysis

The in-depth interviews and focus group discussions were transcribed verbatim and checked against the recording for accuracy, before being translated into English. Inductive thematic analysis was applied to analyze the data. Firstly, researchers read through the entire dataset several times to become immersed and familiarize themselves with the data. All texts were then assigned basic codes to identify important features of the data that are relevant in answering the research questions. Similar codes were classified into categories and further abstracted into broader core themes. The codes, categories and themes were continually compared across transcripts and refined until saturation was reached. In the process of data analysis, interviews of mothers and discussions with hospital staff were considered separately. Two researchers developed the initial coding independently and all researchers contributed to the development of themes from these codes and categories. A qualitative software program, QSR International NVivo 10 (QSR International Pty Ltd, Doncaster, Victoria, Australia), was used to perform analysis of the transcripts.

### 2.4. Ethical Considerations

The study protocol was approved by the local health facilities and the Human Research Ethics Committee of Curtin University (approval number SPH-53-2013). An information sheet explaining the project was given and read to the participants before obtaining their written consent. All participants were informed about their right to withdraw from the study without prejudice. Confidentiality and anonymity of recordings and transcripts were assured throughout the study. The recordings were erased upon completion of the transcription.

## 3. Results

The 50 mothers who took part in the study were aged between 21 and 46 years (median 29 years). The majority of them had graduated from college or university (n = 44) and had a vaginal delivery (n = 31). All mothers had either partially (n = 43) or fully (n = 7) formula feed their most recent child. At the time of interview, 23 infants were aged between 3 and 6 months. Of the 33 hospital staff recruited, the majority were female (n = 30), who had been in practice for more than 10 years (n = 25) and were from the Gynecology and Obstetrics Department (n = 18). Distribution of their positions was: 19 nurses, eight doctors and five midwives.

With regard to why mothers chose to use infant formula, responses given by mothers and hospital staffroom both cities showed commonalities. Table 1 and Table 2 summaries the responses given by mothers and hospital staff, respectively. Several major themes were generated from the in-depth interviews and group discussions, namely, perceived insufficient milk supply, returning to work, beliefs of family and society, and perceived knowledge of infant feeding.

**Table 1:** Reasons for formula feeding: major themes identified by mothers (n = 50).

Major Themes	n (%)
Insufficient breastmilk supply	37 (74%)
Mother has to return to work	8 (16%)
Grandmothers of infant (the mother’s own mother and her mother-in-law) Perception of growth and need for formula	10 (20%)
Yuesao (“confinement lady”) perception of growth and need for formula	9 (18%)
Mothers’ lack of understanding of the benefits of breastfeeding—often think Formula has more nutrition (with Vitamin D, DHA added)	27 (54%)

**Table 2:** Hospital staff responses on reasons why mothers use infant formula.

Major Themes
Insufficient breast milk supply—often believe there are better options for the mother than changing to formula but do not have time or resources; therefore accept the use of formula even though most prefer the mother to continue breastfeeding
Mothers return to work. Because employers do not give support to mothers to breastfeed, it is impossible for mothers to continue to breastfeed
Agreed that the influence of grandparents and Yuesao is very strong. If the baby is given formula, the Yuesao has less work to do as the infant is thought to sleep more
Mothers often get information, such as nutrients added to infant formula, from their peers and do not follow the advice of health workers

### 3.1. Perceived Insufficient Milk Supply

A key reason raised by mothers was the perception of insufficient milk supply. Inadequate milk was reported by thirty-seven mothers who supplemented their infants with formula. The following were four typical ways that mothers used to determine they have “low milk supply”:

- Baby cries after feeding: “*I stopped breastfeeding when my baby was about three months of age.* *My breast* *milk started to dry up at that time. My baby often cried right after feeding*.” (Mother #32)
- No full breasts: “*I began to give my baby infant formula a few days after delivery. I never feel my breasts are full, even after several hours without feeding*.” (Mother #14)
- Only a little breast milk is expressed: “*I had no breast* *milk for the first several days after delivery. Although my milk supply is* *getting more and more, it is still not enough. My baby’s appetite is also becoming larger. Now she needs more than 100 mL per meal, but I can only express 90 mL*.” (Mother #10)
- Baby feeds often: “*My daughter was always crying and unsettled. She became hungry again in a short time after breastfeeding. She looked for food all the time. However, these situations disappeared when I began to give her formula*.” (Mother #42)

Likewise, hospital staff reported that some mothers choose formula feed because of their belief that they were not making enough breast milk:

“*In my work experience, there were many mothers who initiated formula feeding at two or three months postpartum.* *The main reason* *was* *that their milk couldn’t satisfy the baby’s hunger as* *they found the baby got hungry very quickly, was unsettled or crying a lot.*”

(Doctor from Maternal and Child Care Clinic)

Most hospital staff recognized that the “perceived insufficiency” is most likely due to a faulty breastfeeding technique or infrequent feeding. They agreed that it is best to encourage development of an optimal technique in the first hours after childbirth. Many of them, nevertheless, raised the issue of lack of time and resources to provide full lactation support:

“*In addition to assisting mothers with breastfeeding, we have many* *other duties to perform every day. There are too many mothers delivering babies but not enough doctors, nurses or midwives. Sometimes when we are extremely busy, it is impossible to give* *every mother step-by-step instructions on breast* *feeding.*”

(Nurse from Gynecology and Obstetrics Department)

### 3.2. Return to Work

Returning to employment was another reason that affected mothers’ decision to change from breastfeeding to formula feeding or introduce “mixed feeding”. Six mothers indicated that they had to replace some breastfeeds with infant formula during work hours. Another two said that they intended to stop lactating once they return to work. Inadequate facilities, such as the lack of a private room and a refrigerator, as well as limited flexibility of work hours, were mentioned by mothers as obstacles that prevent them from expressing milk at their workplace:

“*It is challenging to find time to express milk whilst working, also not easy to find a place to express milk*.”

(Mother #2)

“*There is no private room for pumping milk at my workplace. I* *don’t want to express my milk in the toilet,* *it is filthy.* *Therefore I go home to breastfeed during lunch* *time and of course after work*.”

(Mother #24)

“*I started to give my baby some formula last week, because I need to go back to work in two weeks. I will then stop breastfeeding totally. I have no other choice. My workplace is far from where I live and there is no breast* *feeding* *room and no refrigerator. It will be too difficult to continue breastfeeding*.”

(Mother #9)

Hospital staff pointed out that many mothers wished to continue breastfeeding after they returned to work. However, the breastfeeding support from employers was far from desirable:

*“Another reason is return to work. It is common in China. I have met many mothers who planned to continue breastfeeding after going back to work. But there are too many barriers to breast* *feeding* *at the workplace. For example, some have to express milk in toilets, and some hide under the office desk when pumping milk. A few workplaces such as hospitals are convenient for mothers to express milk while at work.* *But I believe most employers don’t provide any* *lactation facilities*.”

(Doctor from Maternal and Child Care Clinic)

“*Employers are legally required to give breastfeeding mothers one hour to breastfeed. But I think it is of little help. Mothers can seldom* *bring their baby* *to work. If the mother needs to go home to breastfeed, one hour is obviously not enough. Expressing milk is a good way to continue breastfeeding.* *However, some mothers are too busy to express milk at work and many places* *simply don’t have a private room*.”

(Doctor from Maternal and Child Care Clinic)

Several hospital staff made the comment that few employers are aware of the importance of supporting breastfeeding mothers after they return to employment. The lack of employer support is compromising the effort of breastfeeding promotion. In order to overcome such negative impact, employers should be encouraged to make work environment more breast feeding friendly.

### 3.3. Beliefs of Family and Society

A third common theme generated was the influence of family and society on mothers’ use of infant formula over breast milk. Twenty-eight mothers had sought advice before making their decision to formula feed. When asked who they had approached for help, they (n = 15) responded with more than one source, with a median of 1.5 sources per person. The most frequent request for help was to the maternal mother/mother-in-law (n = 10), followed by friends (n = 9), health professional (n = 9), “confinement lady” (“yuesao”) (n = 9), the husband (n = 5), and other family members (n = 2). One mother reported seeking help from the internet.

Fourteen out of the twenty-eight women received the suggestion of formula feeding from at least one person. The beliefs of maternal mothers and mothers-in-law may have further reduced their confidence in breast milk supply and may serve as stimulation for the introduction of formula:

“*My baby tended to fall asleep quickly at the breast.* *My mother-in-law therefore always said* *my baby wasn’t getting enough breast* *milk and needed to be fed with formula. Gradually, I became doubtful of my milk supply.* *Now* *I am topping up with formula after breastfeeding*.”

(Mother #12)

One mother who introduced formula feeding to her baby at five months postpartum told the researcher:

“*Both my mother and mother-in-law* *think my baby is too small compared to other babies. They often say, for example, the* *neighbor’s baby weighed already 8 kg at 4 months but my baby who is a few days older weighed less. My mum then asked me to eat more food and give my baby some formula*.”

(Mother #43)

The suggestion of a bottle feed from friends and “confinement lady” also contributed to shape a mother’s decision to introduce formula feeding:

“*One of my friends told me if I breastfeed exclusively, my baby may refuse the bottle when I want to wean. In order to help my baby getting used to bottle feeding, I give him some formula every* *day*.”

(Mother #50)

“*Yuesao* *suggested me to feed my baby with some infant formula in the evening, so that he can have a longer sleep at night*.”

(Mother #39)

According to hospital staff, many Chinese mothers face pressure from the older generation who believe that formula feeding is necessary to achieve satiety in the baby:

“*Most families still have only one child in China. All the grandparents focus on the little baby after birth. Whenever the baby cries, the grandmothers and grand* *fathers* *become concerned that the baby remains hungry with breast* *milk.* *On the contrary, if the baby is formula fed, the grandparents usually feel* *more comfortable because they are able to see* *how much the baby has consumed*.”

(Nurse from Gynecology and Obstetrics Department)

Hiring a “confinement lady” has become a trend in the metropolitan areas of China and is regarded as a relief by many mothers of young infants. Nevertheless, hospital staff have expressed concerns that these “confinement ladies”, with no or little formal professional training, may transmit misconceptions about infant feeding to mothers and may encourage mothers to formula feed with the aim of “*making their own work easier.*”

### 3.4. Perceived Knowledge of Infant Feeding

Of the 50 participating mothers, the great majority (n = 43) agreed that breastfeeding is better than formula feeding, while a few mothers regarded formula feeding to be as good as (n = 3) or better than breastfeeding (n = 2). Two mothers stated that they were unsure which one is better.

Better immunity (n = 27), more nutritious (n = 18), convenience (n = 20), easier digestion (n = 7), safer (n = 7), economically cheaper (n = 4), better mother-baby bonding (n = 3), lower the risk of breast cancer (n = 2), and better postpartum recovery (n = 2), were the answers given by mothers when asked about their perceived benefits of breastfeeding. 

Although most mothers regarded breastfeeding as better, twenty-four mothers still mentioned the apparent advantages of formula feeding, such as “more nutritious”(n = 11), “convenience in public” (n = 9) and “less tiring” (n = 6).

### 3.5. Infant Formula is Viewed as More Nutritious than Breast Milk

Among the eleven mothers who regarded infant formula as more nutritious, five women considered assured food safety to be the premise. For example, one mother commented: “*As long as the safety is assured, such as no melamine contamination, the infant formula has better nutrition*.”

Mothers who raised the idea that formula is more nutritious than breast milk rationalized that:

“*My sister is formula feeding only. Her baby gained weight more quickly than my daughter. It is because the nutrients in formula are better*.”

(Mother #4)

“*I was told by doctors at regular check-ups that if I breastfed fully, the baby should take vitamin A or vitamin D supplements. However,* *it is not necessary for formula-fed babies to consume these supplements. So the nutrients contained in formula are more comprehensive.*”

(Mother #29)

“*The infant formula is more beneficial, as it has many additional and essential vitamins and minerals.* *The types and quantities of nutrients are clearly labeled on the packaging*.”

(Mother #40)

Two mothers voiced their opinion that breast milk is better than infant formula only when the baby is very young with a premature digestive system. As the baby gets older, both mothers thought that formula can meet the baby’s need sand allows him/her to grow properly. Moreover, one mother reasoned that she does not eat healthy foods, therefore the nutrition from her milk is not as good as infant formula.

Hospital staff agreed that many mothers accept formula supplement as necessary, due to the perception that breast milk becomes less nutritious as time passes, especially when the milk turns clear and thin or the mother recommences period. 

### 3.6. Formula Feeding is Perceived as Convenient in Public and Less Tiring

Although only one woman reported fatigue and sleep loss as the reason for using formula, twelve mothers linked formula feeding with perceived convenience and/or less tiredness.

Some mothers have the opinion that it is more appropriate to bottle feed in front of strangers than breastfeeding. The latter was viewed as inconvenient by mothers since they have to look for a private room or cover up in public:

“*It’s* *difficult to find a place to breastfeed outside home. I prefer to give my baby a bottle feed when I am down the street. Breastfeeding in public makes me feel uncomfortable*.”

(Mother #34)

Those mothers who found breastfeeding to be more tiring explained that the frequent nursing during day and night deprived their sleep and made them stressed. Formula feeding, on the other hand, would offer them more opportunities to rest:

“*I suffered from extreme sleep loss when my baby was under one month old. I had to breastfeed her every one or two hours, even at night. After starting to give her formula, I feel more relaxed. I can now have a good rest at night*.”

(Mother #15)

Hospital staff group discussions revealed that many mothers desire independence that allows them to have enough sleep for daily activities:

“*Many mothers give formula to help their baby sleep longer at night. The sleep-deprived new mothers are eager to catch up some sleep*.”

(Nurse from Maternal and Child Care Clinic)

“*Formula allows other people to feed the infant. After being confined home for a month after childbirth, some mothers would like their family members help with feeding* *so they can go outside shopping or visiting friends*.”

(Nurse from Maternal and Child Care Clinic)

One doctor from Maternal and Child Care Clinic expressed her concern that the practice of formula feeding at night is becoming a norm:

“*A lot of mothers like the idea of replacing one breastfeed with formula at night.* *They may never consider this as a problem. Some even think this can lead to more breast* *milk supply during the daytime*.”

### 3.7. Sources of Information on Formula Feeding

Most mothers (n = 34) advised that friends and families are a common source of information about formula feeding. Internet (n = 16) emerged as another main source, yet only one mother said she received formula feeding information from hospital staff. 

Indeed, hospital staff described how the increasing access to technology such as social media and mobile applications has allowed mothers to communicate infant feeding ideas with friends and peers online.

“*Just a few mothers ask us questions on formula feeding and the most frequent one they asked is which brand to choose. But we are not supposed to provide a suggestion except for medical reasons. Actually mothers are getting their information more often through the internet. For example, they obtain knowledge on feeding methods from peers by joining some QQ groups. They may also exchange their feeding experience with other mothers via the group chat on We* *Chat.*”

(Doctor from Maternal and Child Care Clinic)

## 4. Discussion

Consistent with previous findings in China [9,10,13], maternal perception of “insufficient milk supply” and returning to employment were the main reasons reported by mothers for introducing infant formula before six months postpartum. The fear amongst mothers that they are not producing enough milk or that the breast milk does not satisfy the infant is often reinforced by the beliefs of maternal mothers and/or mothers-in-law. Our results indicated that, despite most Chinese mother’s perceived breastfeeding as the optimal feeding method, a substantial proportion of them considered bottle feeding to be more convenient, less tiring and more nutritious than breast feeding. The hospital staff perceptions strongly echoed the reasons mothers gave for using formula. This indicates a clear professional understanding of the challenges relating to exclusive breastfeeding within the first six months of life in China.

Perceived “insufficient milk supply” is the most frequently cited reason for the introduction of formula feeding in this study. In fact, only 1% to 5% of women have physiological problems which prevent them from producing enough milk for then newborns [14]. The misconception of inadequate quantity of breast milk may be due to lack of confidence in breastfeeding. A low milk supply can also result from incorrect breastfeeding techniques or infrequent nursing [15]. In the present study, the ways stated by mothers in determining “insufficient milk supply” are inappropriate. Providing mothers with knowledge on the normal physiology of lactation as well as the correct methods of knowing whether the baby is getting enough milk, may help improve their confidence in lactation. Furthermore, mothers should be encouraged to consult health professionals when they encounter any lactation problem. Full support should be given to new mothers immediately after childbirth to establish successful breastfeeding, thereby minimizing or even preventing the use of formula.

With limited flexibility and workplace support, formula supplement seems inevitable for many mothers when they return to work, even though they prefer to continue breastfeeding. Breastfeeding mothers reported difficulties in finding time or place to express milk whist at work. As suggested by hospital staff in this study, raising employers awareness of their obligations to support breastfeeding and perhaps legislating the work environment to be more breastfeeding-friendly are necessary to overcome such barriers. According to Roe and colleagues [16], employers could benefit from supporting breastfeeding mothers because these mothers are less likely to be absent from work due to sick infants.

Family and social support for breastfeeding has been consistently identified as an important factor in infant feeding decisions and practices [17]. Although paternal preference for breastfeeding has been found to be a crucial determinant that influences breast feeding at discharge across different cultures [18,19], the present study suggests that partners had less impact when mothers encountered breastfeeding problems challenging enough to result in their use of a breast milk substitute. Instead, beliefs of maternal mothers and/or mothers-in-law played a strong role in the decision to introduce infant formula. Consequently, mothers and their family members, including the husband and grandmothers, should be targeted in antenatal and postnatal education programs to promote optimal breastfeeding practices.

Social influence from “confinement ladies” also contributed to the choice to formula feed. Employing a “confinement lady” is becoming more popular in urban China to help take care of the mother and newborn in the postnatal period immediately after childbirth. A “confinement lady” is usually an older woman experienced in caring for the special needs of a new mother and her newborn infant according to the traditional confinement practice, which is called “doing the month” or “zuoyuezi”. New mothers may regard the “confinement lady” as an expert in infant feeding and turn to her for advice. Again, encouraging mothers to seek professional advice and increasing their accessibility to certified lactation consultants would prevent unnecessary formula supplementing.

Although most mothers in the study perceived breastfeeding to be healthier than formula feeding for their infants, they appeared to have inadequate knowledge about the health benefits of breastfeeding, especially the benefits for lactating mothers and the long-term advantages for the infant. Without being truly knowledgeable about the health benefits of breastfeeding, mothers may not have the determination to breastfeed and can be easily swayed by the perceived advantages of formula feeding, such as more convenient and less tiring.

Due to greater intake among formula-fed infants and the higher protein levels in formula, formula-fed infants typically gain weight more quickly than their breastfed counterparts between 3 and 12 months of age [20]. Many mothers and their family members believed that the rapid weight gain among formula fed infants resulted from the perceived nutritious ingredients contained in formula. It is thus necessary to inform Chinese mothers and their families about the detrimental impact of gaining weight too fast, which increases the risk of obesity and chronic diseases in later life. In addition, some mothers tended to believe that breast milk is less nutritious after being told by doctors to supplement with vitamin D. It is recommended that health practitioners should always emphasize the importance of breastfeeding and the fact that breastfeeding is still the optimal feeding method even when prescribing vitamin supplements to such infants.

The major strength of this study is the recruitment of both mothers and hospital staff which enabled the comparison of perceptions. Recall errors are unlikely because the mothers’ youngest children were between one and six months of age. Nonetheless, several limitations must be considered when interpreting the results. Firstly, this study included mothers who had partially or fully formula fed their youngest infant, thus the results may not represent the experience and opinions of mothers who chose to breastfeed only. Moreover, China is a large country with its cities in various stages of economic development. Our findings may not necessarily be general sable to the entire population and particularly those residing in rural areas. Finally, the interviews and discussions were conducted in Mandarin and all transcripts were translated into English before data analysis. The use of two experienced bilingual translators minimized any misinterpretation of participant opinions due to translation errors. Future studies, focusing on the impact of advertising and marketing of infant formula on feeding decision, are needed to better understand the role of formula companies in a community with high rates of formula feeding.

## 5. Conclusions

Maternal perception of “insufficient milk supply”, return to employment, influences from family and society, and the lack of truly understanding the health benefits of breastfeeding, were found to be major reasons contributing to the introduction of formula within six months in China. The results enhance our understanding of Chinese women’s perceptions and attitudes towards infant feeding. The findings can also assist health policy makers to develop intervention programs targeting expectant mothers and their families to promote breastfeeding knowledge and practices at an early stage, there by leading to good health for both the mother and her infant.
